# A comprehensive overview of advanced dynamic *in vitro* intestinal and hepatic cell culture models

**DOI:** 10.1080/21688370.2022.2163820

**Published:** 2023-01-21

**Authors:** Filipa Leal, Scarlett Zeiringer, Ramona Jeitler, Pedro F. Costa, Eva Roblegg

**Affiliations:** a BIOFABICS, Rua Alfredo Allen 455, 4200-135 Porto, Portugal; b Department of Pharmaceutical Technology and Biopharmacy, University of Graz, Institute of Pharmaceutical Sciences, Universitaetsplatz 1, Graz, Austria

**Keywords:** Microfluidic devices, gastrointestinal tract, intestine, liver, multi-organ-on-a-chip, bioprinting

## Abstract

Orally administered drugs pass through the gastrointestinal tract before being absorbed in the small intestine and metabolised in the liver. To test the efficacy and toxicity of drugs, animal models are often employed; however, they are not suitable for investigating drug-tissue interactions and making reliable predictions, since the human organism differs drastically from animals in terms of absorption, distribution, metabolism and excretion of substances. Likewise, simple static *in vitro* cell culture systems currently used in preclinical drug screening often do not resemble the native characteristics of biological barriers. Dynamic models, on the other hand, provide *in vivo*-like cell phenotypes and functionalities that offer great potential for safety and efficacy prediction. Herein, current microfluidic *in vitro* intestinal and hepatic models are reviewed, namely single- and multi-tissue micro-bioreactors, which are associated with different methods of cell cultivation, *i.e*., scaffold-based *versus* scaffold-free.

## 1. Introduction

Health care costs are rising globally, especially in the pharmaceutical sector^1^. The reasons for these high costs are manifold; first, the drug development process is lengthy, taking over 12 years, and second, it is risky, as nearly 95% of drug candidates that enter human trials fail.^2^ According to Seyhan, the rate of transfer of new drug candidates from preclinical research to human trials and on to drug approval is about 0.1%.^3^ One explanation for the high failure rate is that translating basic and preclinical research into clinical application in patients is difficult, because using animal models to simulate humans often leads to incorrect results. The lack of efficacy and poor safety profile of new drugs are often not foreseen,^4–6^ owing to the dramatic difference between human and animal anatomy, physiology and pathology. According to a study by Shuler, only about 6% of animal studies are translatable to human responses.^7^ Apart from poor translatability, animal tests raise enormous ethical concerns and are expensive. The result of all these facts is that the knowledge of disease pathobiology is improving and accelerating with new technologies such as next generation sequencing and digital biomarker discovery, while therapeutic advances cannot keep pace for lack of accurate test systems. The European Commission has been pushing the development of alternative test systems, also referred to as 3 R models, *i.e*., Replacement, Reduction and Refinement of animal use in science, for many years to achieve protection of animals but also of human health and the environment. In 2010, the EU Directive 2010/63 clearly set out the full replacement of animal methods as the ultimate policy goal.^8^ According to the report of the European Union Reference Laboratory for Alternatives to Animal Testing (EURL ECVAMA) published in 2020, 9.58 million animals were used for research and testing in 2017, in particular 45% in basic research, 23% in translational and in applied research, as well as 23% for regulatory use, 5% for routine production and 4% for other issues.^9^ To further reduce these numbers, EURL ECVAMA together with funded Horizon 2020 research and innovation projects and collaborative partnerships, has developed, implemented, and validated advanced alternative animal-free technologies. These include *in vitro* methods based on human (stem) cells and artificial tissues, organ-on-chip and microfluidic devices, *in silico* systems and computational models to classify (therapeutic) substances and translate mechanistic understanding of toxicity into safety strategies. EURL ECVAMA is also a representative of the Commission in the OECD Working Party on Hazard Assessment (WPHA). The aim of WPHA is to facilitate and support the hazard assessment of chemicals, especially regarding to the harmonisation of the methods and for testing and evaluation, but also to improve the flow of information on chemicals. In 2020, the WPHA approved two documents, namely an overview of concepts and available guidance with regards to Integrated Approaches to Testing and Assessment (OECD, 2020a) and Guidance on the Characterisation, Validation and Reporting of Physiologically based (PBK) Models for Regulatory Applications (OECD, 2021), with focus on three exposure routes, *i.e*., inhalation, dermal/topical and oral.

The oral route of administration is by far the most common route of drug delivery and is used for both systemic drug delivery and treatment of local gastrointestinal disorders. It is non-invasive and medications can be taken by self-administration, which increases patient compliance while being financially beneficial to the healthcare system.^10^ Despite the clear advantages in drug delivery and the fact that the oral route of absorption has the largest market share at 62%, the human gastrointestinal tract is complex and has a variety of physiological barriers that affect drug delivery. In addition to these challenges, consideration must be given to the physicochemical properties of drug candidates, which are becoming increasingly complex and may exhibit poor solubility and/or permeability and enzymatic stability. To date, there are no animal models comparable to the entire human gastrointestinal tract.^11,12^ Therefore, alternative models capable of mimicking human absorption and first-pass metabolism, as well as serving as cytotoxicity testing platforms, are important to test new compounds rapidly and efficiently and to transfer suitable ones to clinical trials.

In this review, we summarise the current state of knowledge of advanced dynamic *in vitro* cell culture systems that mimic the human gastrointestinal tract. First, a detailed description of the intestine and liver regarding architecture, physiology, organisation and main functions is provided. Next, a general overview of the devices (*i.e*., macro-, micro- and *in vivo* bioreactors) that allow dynamic cultivation with a special focus on microfluidic bioreactors is given and different culture methods are discussed (*i.e*., scaffold based *vs*. scaffold free).

Based on this, current available advanced microfluidic *in vitro* models are presented, and the fields of application are discussed. Finally, linked multi-organ models are outlined and strategies are proposed to overcome limitations of existing models.

## 2. Materials and methods

The literature research for manuscripts related to gastrointestinal dynamic *in vitro* cell culture models was carried out through PubMed, Scopus and Google Scholar. To this end, the search terms or combinations thereof included “dynamic cell culture”, “microfluidic devices”, “organ-on-chip”, “multi-organ-on-chip”, “body-on-a-chip”, “oral-mucosa-on-a-chip”, “stomach-on-a-chip”, “intestine-on-a-chip”, “gut-on-a-chip”, “gut-liver”, “intestine-liver axis” and “gut-liver axis”. Furthermore, the reference section of each article screened was analysed for additional articles dealing with these topics. Patents were excluded from the survey. As the aim of this review article was to address healthy tissue models, articles found for diseased tissue models were not considered.

## 3. The gastro-intestinal (GI) tract

The GI tract is a muscular tube that can be divided into an upper and lower hollow part. The upper part consists of the mouth, pharynx, oesophagus, stomach, and the first part of the small intestine (duodenum). The lower part is composed of the jejunum and ileum and the large intestine (appendix, colon and rectum). In addition, the GI tract includes accessory organs, namely the pancreas, the gallbladder, and the liver. These organs also support the main functions, namely digestion of food, absorption of nutrients (and drug candidates) and excretion of waste products as well as metabolism.^13^ The mechano-physiology of the digestive tract plays another important role. Smooth muscle fibres, which are connected to adjacent muscle fibres via gap junctions, contract due to electrical potential and develop mechanical forces on the mucosal wall. These contractions lead to the development of flow and initiate mixing with various digestive secretions (including important enzymes such as pepsin, pancreatin, bile acids) and transport of ingredients which are then absorbed accordingly (for more details of biomechanics and contractions in the GI tract see Avvari R.K.).^14^ Thereby, transit time is variable and depends on further physiological factors, such as intake of food, age, and gender. Regarding the microenvironment, a series of pH-value changes occur. The pH starts with 6.8 in the mouth, decreases to pH 1.5–2 in the fasted and 3–6 in the fed state in the stomach, rises to 5.8 in the duodenum and increases to 7.4 in the ileum. In the cecum the pH decreases to pH 6 and then rises again to pH 6.8 in rectum.^13^ Because of these variations, the GI-tract displays a variety of protective barriers against mechanical, thermal, chemical and biological effects.^15^

During or after absorption, drugs are metabolised – either directly in the intestinal wall or in the liver. The liver plays not only an important role in the metabolism of substances but also in detoxification, conjugation and activation.^16–18^

## 4. Architecture, physiology and organisation of the small intestine and the liver

**Figure 1.**
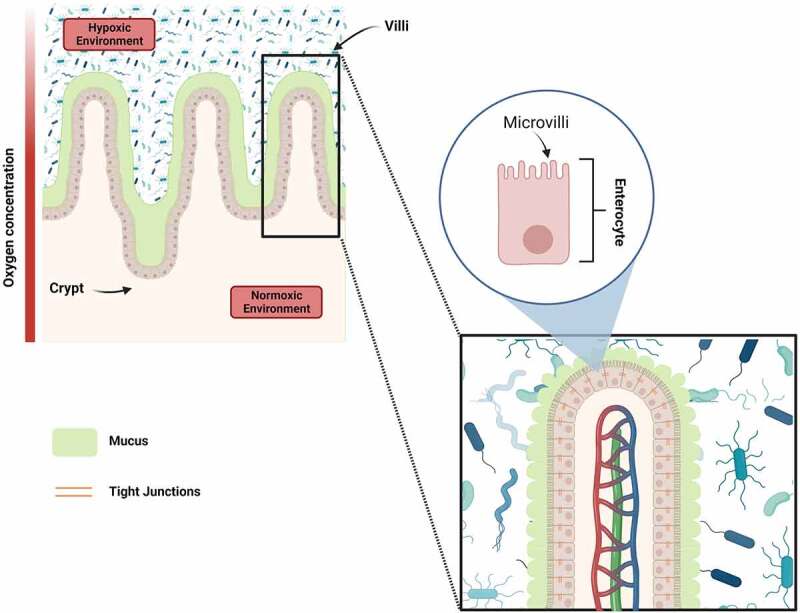
Structure of the small intestine including villi and crypts considering the hypoxic and normoxic environment (oxygen concentration). In particular, enterocytes (90%), which are connected via tight junctions, show microvilli at the luminal side that increase the surface area and are covered with mucus (5% goblet cells). Created with biorender.com.

The small intestine is the main location where absorption occurs. Because of the formation of villi and microvilli, the surface area is rather large and well supplied with blood vessels. The epithelium is composed of different cell types with columnar shapes and tight cell-cell junctions, covered by a mucus layer. The columnar shape is established by the formation of the typical crypt-villus units (Figure 1). Crypts are invaginations of the intestinal epithelium that primarily accommodate proliferating cells, enabling the renewal of the epithelial cells.^19^ In addition, villi are finger-like structures whose functions include lumen sensing, digestion, absorption, secretion, and immune defense.^20^ The epithelial cells themselves form microvilli, which are actin-based membrane features. Their functions include absorption, secretion, mechano-transduction and increase of the surface area leading to a greater interaction with nutrients and drugs.^21^

The intestinal epithelium consists of a cell monolayer composed of 90% absorptive enterocytes and 5% of mucus-producing goblet cells. The remaining percentages are divided among enteroendocrine cells, tuft cells and Paneth cells. Absorptive enterocytes secrete enzymes that enable digestion and absorption of nutrients from the intestinal lumen. Secreted mucins from goblet cells form a mucus-layer throughout the intestine, which acts as a physical barrier and therefore protects the intestinal epithelium from food, digestive secretions and microbial harm. In contrast, the mucin layer also enables the establishment and maintenance of the typical gut microbiota. Glycans, derived from mucins, serve as an energy source for the microbial ecosystem.^22–24^ The epithelial cell layer is bordered by an extracellular matrix (ECM), which consists of various proteins, mainly collagens, and comprises blood vessels, immune cells and enzymes. Transmembrane receptors, such as integrins, mediate the contact between ECM and epithelial cells. This interaction enables the cells to react to external factors, *e.g*., flow and motility of the intestine and the resulting transport of faeces. Both cause shear stress of the intestinal epithelium, which leads to an activation of the receptors and, as a consequence, to a biological response.^24,25^

The hepatic tissue consists mainly of cuboidal shaped hepatocytes, which are highly differentiated parenchymal cells of epithelial origin and are largely responsible for the typical liver function, which include synthesis and secretion/excretion of bile and plasma proteins (*e.g*., albumin, fibrinogen and transferrin), gluconeogenesis, amino acid decomposition, urea synthesis and storage of glucose, iron and vitamin. Moreover, hepatocytes are the main responsible for detoxification and phase I (carried out by cytochrome P450 (CYP450) enzymes) and phase II (carried out by transferases) metabolism of xenobiotics and drugs.^2,5,26^ The second largest liver cell population comprises non-parenchymal cell types including Kupffer cells (KCs; macrophages), stellate cells (also known as Ito cells), liver sinusoidal endothelial cells (LSECs), biliary epithelial cells and cholangiocytes (both lining the intrahepatic and extrahepatic bile duct system).^26–29^

Each cell type has different essential tasks such as the creation of a tubular structure allowing mass and gas exchange between blood and tissue (*i.e*., LSECs), storage of vitamin A and lipid droplets (stellate cells), adjustment of blood microcirculation (stellate cells), collagen production, secretion of ECM molecules, deposition of the matrix in Disse space (stellate cells) and phagocytosis, as well as endocytosis of xenobiotics, toxins and particles (KCs). Therefore, they are involved in the maintenance of tissue structure, modulation of hepatocyte phenotype, and regulation of liver responses following metabolic and toxic stimuli.^6,10,26^

**Figure 2.**
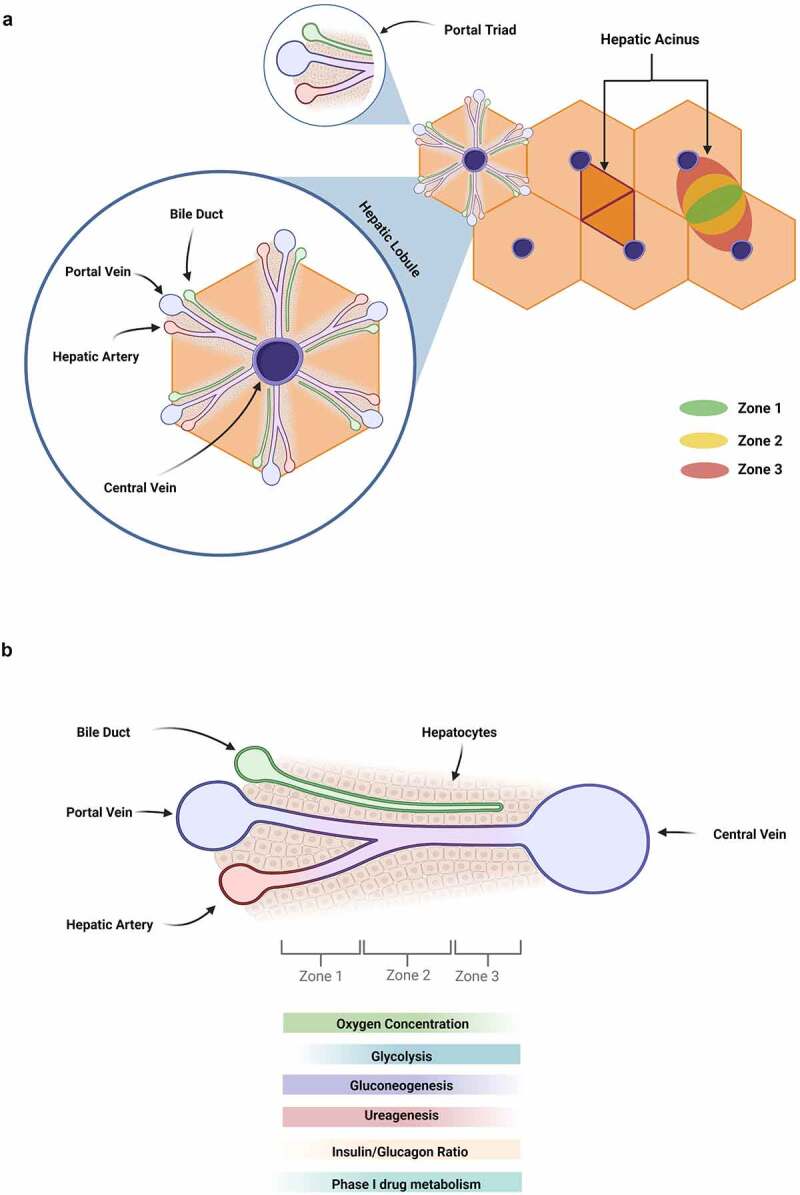
Structure and resulting “metabolic zonation” of the liver including (a) lobules and acini. Each lobule has the central vein in the middle from which sinusoids radiate to the corners where the portal triad is situated. (b) The hepatic sinusoid comprises the portal triad, which encompasses the portal vein, hepatic artery and bile duct surrounded by cuboidal shaped hepatocytes. The zoning of the lobules results from the oxygen, nutrient and hormone gradient. Created with biorender.com.

The human liver tissue is further divided into two main lobes, which in turn consist of several functional units – the hepatic lobules. Each lobule has a hexagonal prismatic morphology, with the central vein in the middle from which sinusoids radiate to the corners where the portal triad is situated (Figure 2). The portal triad encompasses the portal vein, hepatic artery and bile duct, which are surrounded by hepatocytes.^30,31^ The arrangement of the different cell types and ducts in the lobules creates a dense sinusoidal network that enables effective oxygen transport and exchange of nutrients, as well as high metabolic reactions between blood and tissue.^31,32^

The cellular structure of the lobule and the accompanying cellular functionalisation result in a zone-dependent partitioning of the tissue, which is referred to as “metabolic zonation” (zone 1 to zone 3, Figure 2a). The zoning results from a further subdivision of hepatic lobules into acini. Each lobule has six hepatic acini that are defined by the axis of the terminal venule, the terminal hepatic arteriole and bile duct branch of the portal area (Figure 2a). In zone 1 and 3, hepatocytes surround the portal tracts and the central veins, respectively. Zone 2 hepatocytes are located between these two zones.^33^ Along the zones there is an oxygen, nutrient and hormone gradient, with the highest concentrations present in zone 1, which then continue to decrease in the other zones. However, zone 3 is abundant in metabolites and CYP450 proteins (Figure 2b).

Understanding the complex cellular architectures and the diverse functions and organisation of the gut and liver, respectively, are important for their adequate *in vitro* modelling and must be addressed accordingly, as discussed in more detail in the next sections.^34^

## 5. Advanced *in vitro* systems

Traditional static two-dimensional (2D) and three-dimensional (3D) *in vitro* models, while providing a robust and cost-effective alternative to the expensive, ethically questionable and not always transferable animal experiments, have numerous limitations. For instance, the models are usually suitable for short-term cultivation as the viability of the cells is timely limited and cultured cell lines or primary cells can lose their functionality due to dedifferentiation. Moreover, the complex microstructure of tissues cannot be fully reconstructed, and cells in static 2D cultivation may exhibit differences in mechanical and chemical characteristics compared to those *in vivo*, due to lack of ECM, intracellular interactions/signalling and fluid dynamics. These factors play an essential role in cell differentiation, viability, expression of genes and proteins, proliferation, polarity, absorption and drug metabolism.^35,36^ Therefore, advanced cell culture systems that allow physiological replication of human tissues are increasingly needed to study drug effects as accurately as possible.

**Figure 3.**
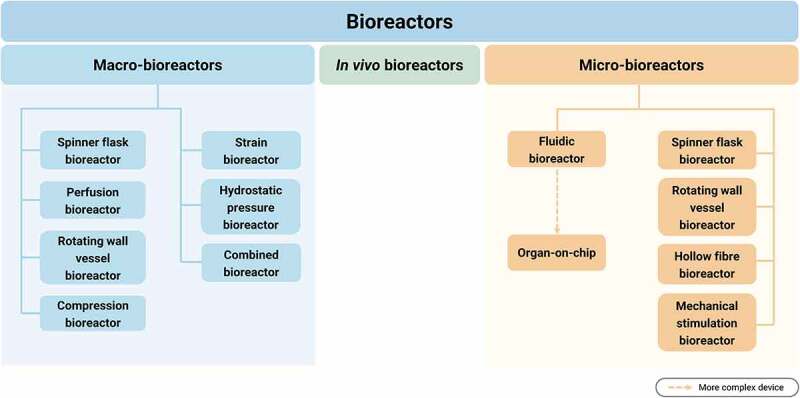
Overview of the distinct types and subtypes of bioreactors.

Cultivation under dynamic conditions offers a promising strategy for overcoming the aforementioned limitations. Various (sub)types of bioreactors can be used for this purpose, which are divided into *in vivo*, macro- and micro-bioreactors and are shown in Figure 3. *In vivo* bioreactors use the body of an organism as a bioreactor to incubate a biomaterial or an immature tissue engineering construct. Here, the regenerative capacity of the body is used to form neovascular and neural tissue and to promote the immune system compatibility.^37^ Macro-bioreactors are used predominantly to develop functional tissue for *in vivo* implantation, and to overcome challenges of conventional cell seeding methods. Within the macro-scale bioreactors, different sub-types of bioreactors can be adjusted depending on the tissue requirements. These subtypes include spinner flask bioreactor, perfusion bioreactor, rotating wall vessel bioreactor, compression bioreactor, strain bioreactor and hydrostatic pressure bioreactor, as well as combinations of these subtypes.

**Figure 4.**
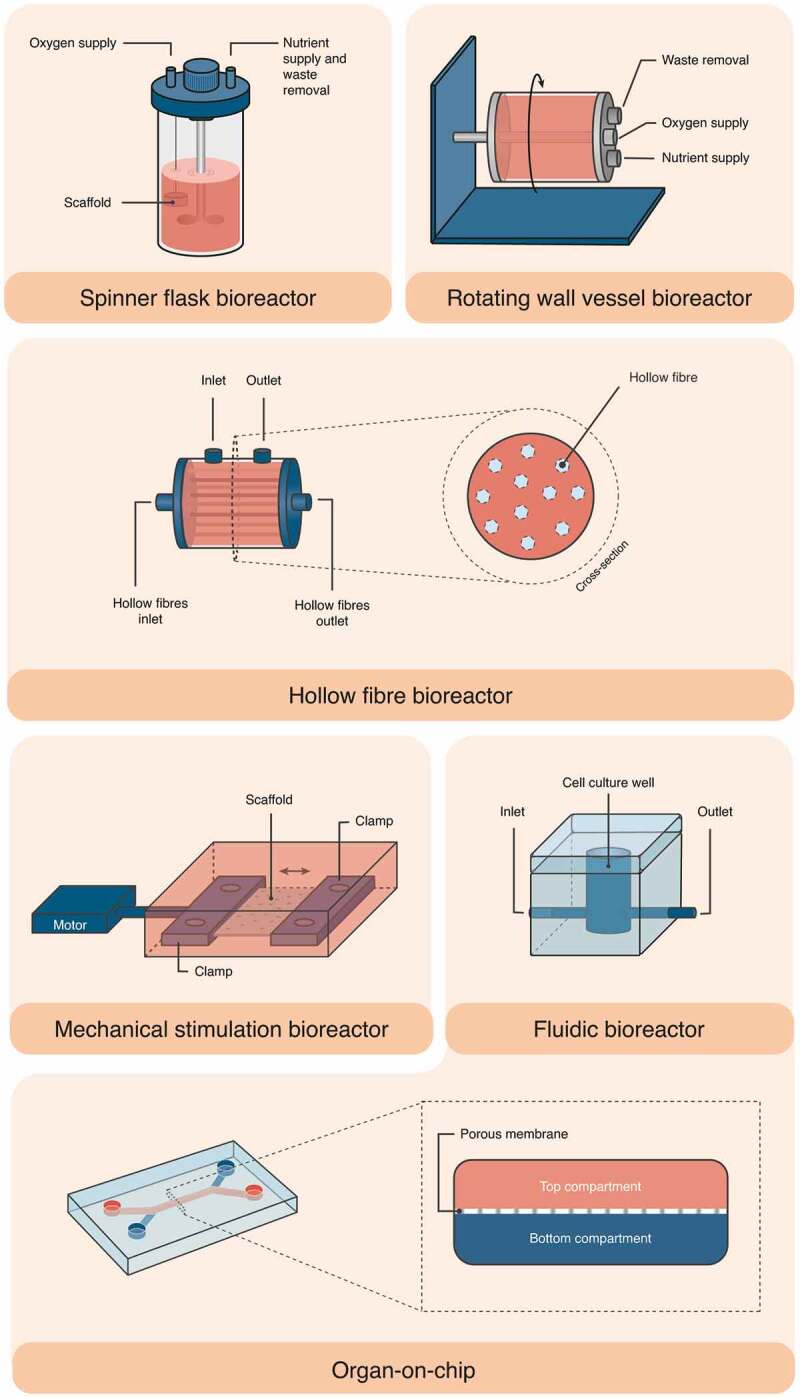
Schematic illustration of the structure of various micro-bioreactor subtypes, i.e., spinner flask bioreactor, rotating wall vessel bioreactor, hollow fibre bioreactor, mechanical stimulation (stretching) bioreactor, fluidic bioreactor and organ-on-chip.

Although tissue engineering is more associated with regenerative medicine, it has also been used as a method to develop physiological models with various functions, in particular, for drug screening and testing, disease investigation, clinical prognostics and diagnostics, and personalised medicine. To fulfil these functions and enable optimal growth and metabolic activity of cells, specialised small-scale systems called micro-bioreactors have been developed. This technology can accurately simulate the physiological environment *in vivo* and enable the development of microenvironments in the cellular size range.^38^ The process can be controlled, is reproducible and reliable, and the required space and incubator volume is low, requiring lower amounts of samples, reagents, and consumables. It is scalable, easy to handle and monitor,^39^ allows the study of several parameters that affect cell functions, and provides more sensitive analyses due to the surface-to-volume ratio.^40^ Micro-bioreactors can be divided into stirred systems or spinner flasks, rotating wall vessel, hollow fibre micro-bioreactors, micro-bioreactors for mechanical stimulation and microfluidic bioreactors. For a better understanding, their schematic structures are depicted in Figure 4.^41,42^

The spinner flask system is a simple bioreactor with scaffolds anchored inside a flask through needles or wires. It uses a stir bar to generate fluid flow and provides gas exchanges through side arms.^43,44^ The rotating wall vessel is a cylindrical bioreactor that maintains cells in suspension by rotation in its horizontal axis, generating low shear forces and high mass transfer.^45^ Despite this, inhomogeneous cell distribution and extracellular deposition occur, which may interfere with cell attachment and matrix deposition on the scaffolds. Both bioreactors are unable to adequately perfuse culture medium across the scaffold, lacking proper stimuli to the cells and not providing nutrients through the entire scaffold.^46^ Hollow fibre micro-bioreactors consist of several semi-permeable fibres fixed into a device on which cells are cultured on the outer side. Medium is perfused through the lumen allowing nutrients, gas and metabolites to be diffused through the membrane. This provides the necessary conditions for cell growth and proliferation.^47,48^ Mechanical stimulation of micro-bioreactors is applied by stretching or compressing the cell-based construct/scaffold or tissue, in order to mimic the forces to which cells are exposed in the native tissues.^41^ Using microfluidic systems, a precise and reproducible control of the required environmental conditions (*i.e*., temperature, pH and oxygen) is enabled. Moreover, the introduction of dynamic flow conditions (*i.e*., circulation of culture medium within the device) allows not only the supplementation of cells with nutrients and the removal of waste metabolites, but also the replication of mechanical cues such as shear stress.^26^ When mentioning microfluidic bioreactors, one cannot leave aside organs-on-chips. It is not easy to distinguish between microfluidic bioreactors and organs-on-chips, as their definitions are not standardised. Organs-on-chips are microfluidic cell culture devices capable of reproducing the physicochemical microenvironment of a human organ or tissue using smaller volumes of endogenous/physiological fluids. They are more sophisticated systems than microfluidic bioreactors as they incorporate elements from classical bioreactors and novel technologies such as sensors, microfluidic components and cell scaffolds.^41^ Such systems have recently been shown to be particularly useful for preclinical drug testing, toxicity testing and in biomedical science for the study of diseases.^49–51^ Furthermore, they can support 3D cultivation by using multi-cell aggregates, microspheres and cell encapsulation in order to better recapitulate the *in vivo* environment.^52^ A study conducted between 2015 and 2019 on the type of micro-bioreactors most used in research revealed that fluidic micro-bioreactors (also referred to as microfluidic bioreactors/systems/devices and/or organs-on-chips) are the most prominent ones and are, therefore, addressed in this review.^41^

## 6. Cell cultivation methods in microfluidic devices

**Figure 5.**
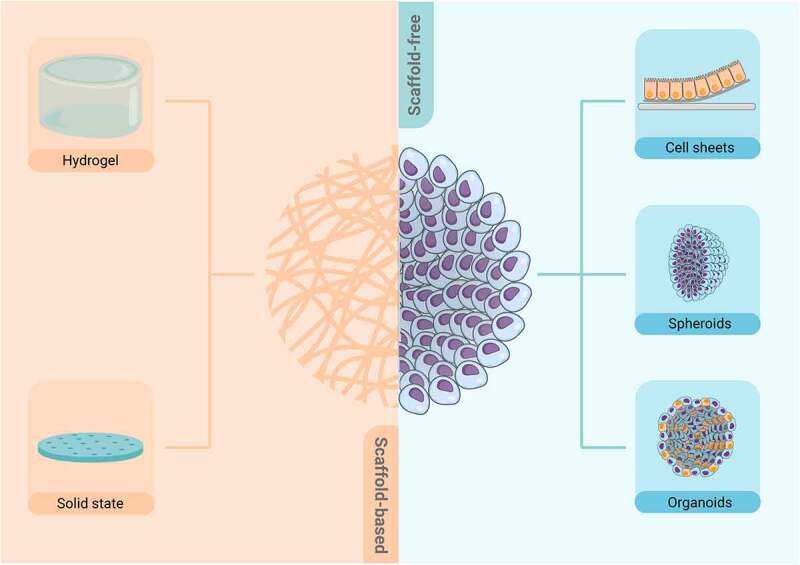
Cultivation methods in microfluidic devices divided into scaffold-based and scaffold-free. Scaffold-based methods, on the left, use biomaterials in hydrogel or solid form as a structural base to support cells during tissue growth and maturation. On the right, scaffold-free techniques, which do not require artificial scaffolds for tissue formation, are presented.

Cultivation methods in microfluidic devices can be categorised into scaffold-based and scaffold-free (Figure 5). Scaffold-based methods including hydrogel- and solid-state-scaffolds provide a physical support on which cells can aggregate, proliferate and migrate.^35,53^ The used matrices should be biocompatible and mimic the chemical and physical characteristics of *in vivo* tissue. Hydrogels are the most widely used scaffold-based candidates because they can mimic the ECM while allowing cytokines and growth factors to pass through the matrix. Another advantage is the variety of hydrogels available, which can be natural or synthetic and have different mechanical and chemical properties.^53^ Matrices made from naturally derived polymers, for example, have the advantage that they possess cell adhesion sites and can be used in combination with other polymers to achieve certain therapeutic, regenerative and mechanical properties.^54^ In contrast, solid-state scaffolds can be produced with ceramics, metals, glass, and polymers and enable the positioning of cells in a controllable and reproducible way. Thereby, porous polymer scaffolds, *e.g*. produced by electrospinning can be designed with the desired porosity, pore size and surface area to volume ratio.^35,55,56^ The use of scaffold-based systems also provides sufficient stability to exert shear stress or simulate peristaltic movements. Their adaptability to physicochemical properties enables the application of mechanical stimuli, like flow conditions or cyclic strain mimicking the intestinal physical microenvironment. In the case of polydimethylsiloxane (PDMS) membranes, for example, peristaltic movements can be simulated by applying a vacuum resulting in tensioning and stretching of the membrane.^57,58^ In addition to the material of the matrices, the design of the systems should be considered. Specific designs such as reported by Trietsch *et al*., allow the cells to form blood vessels and tubes^59^ or provide a structural basis in form of, *e.g.*, the crypt-villus axis of the intestine.^58,^ This in turn enables a simulation that is more in line with the physiological conditions.

Although scaffold-based systems provide structure and stability for the application of biomechanical stimuli, cells often require a special underlying substrate to adhere properly. Therefore, many scaffolds, especially solid-state and porous polymer scaffolds, require coating with ECM or collagen products to achieve adequate cell adhesion. However, this coating can cause blocking of the micropores of the scaffolds, resulting in limited and altered diffusion and permeability. Another disadvantage in using scaffold-based systems is the possible influence on the absorption rate through the scaffolds or, especially for hydrogels, batch-to-batch variations.^61,62^ Furthermore, scaffold-based techniques can induce an immune response in the body or be toxic to surrounding tissues and therefore scaffold-free techniques have been developed as an alternative method.^63^

Scaffold-free techniques include systems such as spheroids, organoids and cell sheets. Spheroids are heterogeneous self-organising cell aggregates that can be formed from a wide range of cell types (*e.g*., tumour tissue, embryoid bodies, nervous tissue, or mammary glands). They are able to mimic the properties of solid tissues, establish geometric and ideal physiologic cell-cell and cell-ECM interactions, form their own ECM components, remove metabolic waste and develop gradients of oxygen, nutrients and signalling molecules.^31,35^ However, difficulties can arise in spheroid cultures when attempting to produce spheroids with uniform size and low cell number, or when trying to control the specific ratio of cell types in the co-culture. In turn, organoids are 3D cell aggregates derived from primary tissues or stem cells that are able to self-organize and recapitulate the major functions of native tissues. They are also capable of mimicking the complex spatial morphology of a differentiated epithelium to establish cell-cell or cell-ECM interactions.^64^ Nonetheless, this 3D system has several limitations, as some of the organoids replicate only in the early stages of organ development, important cell types are missing, and the structure and functions of an organ cannot be fully mimicked due to the absence of blood vessels.^65^ Despite these limitations, organoids originate more tissue level phenotypes with higher tissue complexity than spheroids.^31^ Finally, another promising technique for tissue engineering is cell sheet engineering. In this method, cells attach and grow on a temperature-responsive polymer culture dish surface. Once the temperature decreases to values below 37°C, the polymer surface changes due to hydration, so that the cells are no longer able to adhere to the surface and are harvested as a single, contiguous cell sheet. Hence, intact cell-cell junctions can be obtained, and the sheets can be stacked on top of each other to obtain a 3D structure. This has the advantage of achieving high cell density and high grafting efficiency with variable structure. Additionally, long-term engraftment is provided, and cell proliferation and adhesion can be improved.^66^ However, it needs to be noted that the lack of vascularisation can cause necrosis of the cell sheets. In addition, a surface coating is required to prevent the cells from clustering and to form an evenly distributed layer of cells. The decrease in temperature and the forces applied to cell sheets during the process is also a main issue since cell sheets are shrinking in size, which in turn has to be considered during cell seeding. This leads to a high cell number, which is required for the cultivation of cell sheets.^67^ Most of the scaffold-free methods also have in common that they lack the necessary stability to apply shear forces to the systems. Therefore, it is not possible to simulate flow conditions or cyclic mechanical deformations resembling physiological conditions on the cell models.^68^ Another common disadvantage of scaffold-free techniques is the lack of standardisation of the used experimental procedures and thus the poor reproducibility, as elaborated by Raghavan *et al*.^62,69^ Additionally, it was found that the investigation of the cell proliferation in organoids and spheroids is difficult to perform due to limited access to the inner side of the cell structure. Problems with the visualisation because of limited diffusion through thick cell layers are also described. More detailed information can be found in the review by Temple *et al*.^62^

## 7. Microfluidic *in vitro* models of intestine and liver

Predictive assessment of drug absorption, metabolism, toxicity, intestinal and hepatocellular functions, and organ-specific pathologies requires fully functional models that recapitulate the spatial and temporal tissue microenvironment.^34,70^ Furthermore, drug-induced liver injury needs to be detected early, as it is not only a major health risk, but also the main reason for drug failure or withdrawal of the already approved drugs.^71,72^

Currently, all models available consider two fundamental aspects: i) identification of suitable cell sources and ii) design of systems that allow the culturing of selected cells while maintaining the full functionality of each cell line. Regarding the liver, a distinction is made here between *in vitro* models for the short-term investigation of drugs and so-called long-term models, which are used for longer experiments to investigate for example, the chronic toxicity of drugs (several days to weeks).^73^

### 7.1. Microfluidic intestinal models

To replicate the intestinal epithelium, immortalised cell lines such as human colorectal adenocarcinoma (Caco-2) cells and the mucus-secreting human colon cancer HT29-MTX cells are commonly used.^74,75^ Caco-2 cells are capable to spontaneously differentiate into enterocyte-like cells, acquiring upon confluence a characteristic apical brush border with microvilli, forming tight junctions and expressing enzyme activities and transporters typical of enterocytes.^76^ These characteristics are essential to accept this cell line as the standard cell model for drug intestinal permeability studies.^61,77–79^ HT29-MTX cells, in turn, are able to modulate the second most abundant cell type of the intestine since they can differentiate into goblet-like cells and secrete small amounts of mucin 2 (MUC-2) during cultivation.^80,81^ None of these cell lines alone exhibits the phenotype and functions of the native gut, but the co-culture provides a model that acts as a barrier to the uptake of certain components.^82^ Moreover, tissue-tissue interfaces between the vascular endothelium and the intestinal epithelium are essential for tissue functionality.^83^ Endothelial sources such as human umbilical cord vein endothelial cells (HUVECs), human intestinal microvascular endothelial cells (HIMEC) and immortalised human vascular endothelial (EA.hy926) cells have been used.^84,85^ Other systems also included human peripheral blood mononuclear cells (PBMCs) and human CD4 + T cells as representatives for immune cells.^86,87^

**Figure 6.**
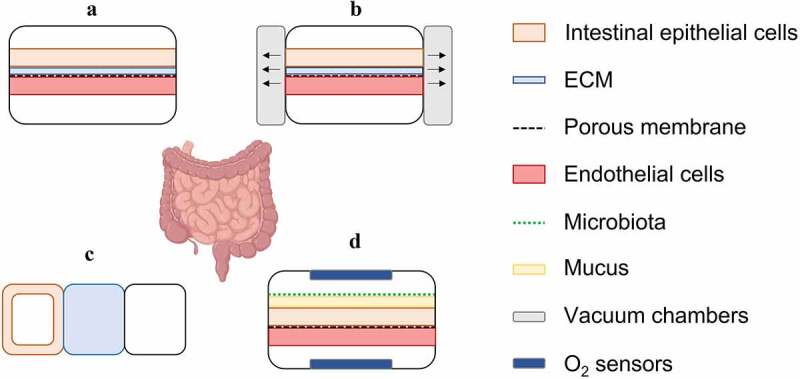
Exemplary schematic cross-sectional illustrations of microfluidic intestinal models. (a) The basic design consists of two channels divided by an artificial membrane coated with ECM (based on Thuenauer *et al*.^88^). (b) By implementing two vacuum chambers, peristalsis can be mimicked (based on Grassart *et al*.^57^ and Kim *et al*.^58^). (c) The three channel model developed by Trietsch *et al*.^59^ consists of an intestinal tubule, a channel with extracellular matrix gel and a perfusion lane. (d) The schematically illustrated model of Jalili-Firoozinezhad *et al*.^89^ comprises endothelial cells in the basolateral compartment, which is separated from the apical compartment by a porous membrane. There, bacteria are in co-culture with intestinal tissue under hypoxic conditions monitored by oxygen sensors (based on Kim *et al*.^90^ and Jalili-Firoozinezhad *et al*.^89^).

The designs of intestinal microfluidic devices are illustrated in Figure 6anda detailed overview of the *in vitro* models considering the aforementioned cell lines and physiological conditions is given in Table 1. The basic design for culturing intestinal cells consists of at least two channels, an upper channel as the apical side, a lower channel as the basolateral side, and a porous membrane. The artificial membrane separates the channels and can be coated with collagen or Matrigel® to simulate the ECM prior to cell culturing on the apical side^88^ (Figure 6a).

**Table 1.** Comprehensive overview of the *in vitro* intestinal microfluidic models considering the cultivation method, cell types used, presence of microbiota and peristalsis-like motions, culture period and application.

Scaffold-based *vs*. scaffold-free	Cell types	Mono- *vs*. Co-culture	Microbiota (Yes/No)	Peristalsis-like motions (Yes/No)	Short-term *vs. long-term*	Application	Reference
Scaffold-based	Caco-2 cells	Mono-culture	No	No	Short-term (up to 4 days)	Permeability studies; cell differentiation studies; barrier integrity assessment.	^59^
Scaffold-based	Caco-2 cells	Mono-culture	No	No	Short-term (up to 5 days)	Barrier integrity assessment; cell differentiation studies; permeability studies; drug testing of a lipophilic prodrug.	^91^
Scaffold-based	Caco-2 cells	Mono-culture	No	No	Long-term (14 days)	Permeability and cell differentiation studies; drug screening.	^92^
Scaffold-based	Caco-2 cells	Mono-culture	No	No	Short-term (3 days)	Permeability studies; cell differentiation studies; toxicity studies; barrier integrity assessment; pathogenesis; drug absorption studies.	^93^
Scaffold-based	Caco-2 cells; human intestinal subepithelial myofibroblasts (ISEMFs)	Co-culture	No	No	Short-term (up to 5 days)	Barrier integrity assessment; cell differentiation studies; proliferation studies; intestinal absorption evaluation; drug testing.	^94^
Scaffold-based	Caco-2 cells	Mono-culture	No	Yes	Short- to long-term	Human intestinal physiology studies; uptake studies; drug screening and development.	^58^
Scaffold-based	Caco-2 cells	Mono-culture	No	Yes	Short-term (up to 6 days)	Permeability studies; viability studies; cell differentiation studies; pathogenesis; host-pathogen interactions.	^57^
Scaffold-based	Caco-2 cells	Mono- and co-culture	Yes (*E. coli*)	No	Long-term (up to 21 days)	Host-microbiota interactions; differentiation studies; viability studies; drug testing.	^95^
Scaffold-based	Caco-2 cells	Co-culture	Yes (LGG; complex microbial community)	No	Short- to long-term	Host-microbiota interactions studies; permeability studies; viability studies; cell-cell interaction studies.	^96^
Scaffold-based	Caco-2 cells, HUVECs; PBMCs; primary human macrophages	Co-culture	Yes (LGG; *C. albicans*)	No	Short-term (up to 7 days)	Host-microbiota interactions studies; permeability studies; microbial communication mechanisms, immune cell activation and pathogenesis studies.	^97^
Scaffold-based	Caco-2 cells; non-cancerous colonic (CCD-18Co) cell line; primary human CD4 + T cells	Co-culture	Yes (LGG; *Bacteroides caccae*)	No	Short-term (7 days)	Barrier integration assessment; host-microbe interaction studies.	^98^
Scaffold-based	Caco-2 cells and HIMECs; human primary intestinal epithelial cells	1^st^ model: co-culture of Caco-2 cells, HIMECs with obligate anaerobe bacteria or complex microbiota; 2^nd^ model: co-culture of human primary intestinal epithelial cells with human gut microbiota	Yes (*Bacteroides fragilis*; healthy human gut microbiota originally collected from infants)	Yes	Short- to long-term (up to 12 days)	Differentiation studies; paracellular permeability studies; toxicity studies; barrier integrity assessment; drug toxicity.	^89^
Scaffold-based	Caco-2 cells	Mono-culture	Yes (LGG)	Yes	Short- to long-term (up to 12 days)	Barrier integrity assessment; differentiation studies; paracellular permeability studies; host-microbiota interactions studies; viability studies of cells and microbes; studies on the mechano-regulation of intestinal function.	^90^
Scaffold-based	Human intestinal epithelial cells derived from organoids established from endoscopic biopsies of living human intestine; HIMECs	Co-culture	No	Yes	Short- to long-term (12 days)	Paracellular permeability studies; barrier integrity assessment; cell differentiation studies; evaluation of drug delivery and therapeutic efficacy or toxicity.	^99^

The first parameter that should not be disregarded to be implemented into a model is shear stress as the peristalsis and luminal flow affect the characteristics of the cells and the tissue. Studies showed that applied flow conditions and shear stress are responsible for changes in the morphology of the intestinal epithelium. These include the formation of a polarised epithelial cell monolayer with basal nuclei and columnar shape,^90,97^ a faster differentiation of Caco-2 cells and hence the formation of microvilli^91^ as well as an enhancement in barrier integrity.^58,90,97^ All these factors lead to an increase in surface area and in metabolic activity.^58,59,89–91,93,97,99^ Since the intestinal movements are not only described by flow conditions, researchers have reconstructed the intestinal peristaltic movements that propel the chyme towards the colon.^100^ These movements were simulated by applying vacuum in two chambers attached to the microfluidic device, which leads to negative pressure and thus causes stretching and tensioning of the membrane (see Figure 6b). This results in a fast organisation into the villi-crypt structure and a polarised and well-differentiated cell layer.^57,58^ For example, Grassart *et al*. demonstrated the need for replicating peristalsis in their study evaluating the infectivity of the human pathogen Shigella.^57^ It was found that the infection was significantly increased, and minimal bacterial load was sufficient to infect enterocytes from the apical side. This was associated with a loss of barrier integrity and a colonialisation of Shigella in epithelial crypt-like invaginations, which occur under dynamic and peristaltic conditions. Moreover, mechanical forces augmented the invasion of Shigella. As the data were in good agreement with *in vivo* observations, the established intestine chip model provides a useful tool for testing effects of pathogens on intestine tissue under physiological conditions.

Apart from the flow rate and peristalsis, the architecture of the equipment (*e.g*., geometry of micro-channels, height of chambers, etc.) also affects the flow characteristics and thus the mechanical stimulation, proliferation and spreading of cells.^95,101^ As an example, more advanced systems consist of three channels converging in the centre, in which two capillary pressure barriers, called phaseguides, pattern the ECM gel^59^ (see Figure 6c). The ECM allows the mimicry of biochemical and mechanical environment^102^ and at the same time increases cell adhesion.^58,59,89–91,97,98,99,103^ Culture media is perfused on both sides of the ECM gel, with one side containing Caco-2 cells lining the channel and the gel surface, forming a tubular shape. It was found that Caco-2 cells cultured on this device exhibited tight junctions, brush border and dome formation and showed better barrier integrity than on Transwell® models.^59^ The mucus layer that covers the enterocytes is incorporated in intestinal models by using goblet cells^74^ or by stimulating Caco-2 cells to produce MUC-2^98^. Regarding the latter one, differentiation and MUC-2 production are modulated by fluid flow.^90,93^ The mucus layer remains intact under dynamic conditions and has been shown to increase cell viability in the presence of bacteria.^96^

Another factor that should not be neglected in the development of intestinal *in vitro* models is the intestinal microbiota. This consists of a colony mainly composed of bacteria but also archaea, viruses and eukaryotic microbes that maintains an interdependent relationship with the human body under healthy conditions.^104^ These organisms are involved in metabolic processes, interact with the immune system and hence protect against pathogens and affect physiological functions.^105,106^ To mimic the intestinal microbiome *in vitro* and understand the impact on the architecture and functions of the intestinal epithelium, microfluidic intestinal models were exposed to bacteria, such as *Lactobacillus rhamnosus GG* (LGG) and *Escherichia coli*. Compared to studies performed under dynamic conditions, static co-cultivation caused a significant decrease in cell viability (~ 30%)^90,95^ and loss in barrier integrity.^90^ Since most microorganisms in the gut are anaerobes, they require a luminal oxygen content of less than 0.5 %, thus, the oxygen gradient must also be taken into account.^89^ To ensure satisfactory conditions for both epithelial cells and bacteria, Jalili-Firoozinezhad *et al*.^89^ equipped the model developed by Kim *et al*.^90^ with oxygen sensors for real-time monitoring and co-cultured Caco-2 cells and LGG in the apical compartment under hypoxic conditions. Furthermore, they included endothelial cells in the basolateral compartment (Figure 6D). This set-up enabled the supply of oxygenated medium through the porous membrane to the epithelial cells from the basolateral side, maintaining cell viability and an intact barrier integrity.^89^ A different approach considering the oxygen gradient but excluding endothelial cells was developed by Shah *et al*.^98^ Simulation of immune responses is another important aspect for the physiological replication of intestinal models comprising epithelial and endothelial cells and bacteria. Maurer *et al*. established a two-channel model to demonstrate physiological interactions between cell layers in an immunocompetent environment.^97^ For this, endothelial and innate immune cells were cultured in the upper channel and intestinal epithelial cells and bacteria were cultured in the bottom channel. No inflammatory responses were induced by bacteria (LGG); however, in an endotoxemia model, Toll-like receptor 4 agonist lipopolysaccharide (LPS) and *Candida albicans* induced inflammation, thereby showing an *in vivo*-like immunocompetent environment. The latter model showed that the presence of LGG improved cell viability and limited the growth of *Candida albicans*, which clearly demonstrated beneficial effects of the microbiota in intestinal *in vitro* cell culture models.^97^

Microfluidic systems can also pave the way to personalised medicine by providing the possibility to culture biopsy-derived human tissues enabling the reproduction of individual patient cell characteristics.^89,99^ Jalili-Firoozinezhad *et al*. made some progress by co-culturing isolated primary ileum cells and fresh gut-microbiome derived from infant stool samples. Multilineage differentiation, villus formation and mucus production was obtained.^89^

Apart from scaffold-based cultivation strategy, microfluidic systems also enable scaffold-free cultivation of organoids and spheroids and/or both. Kasendra *et al*. cultivated human intestinal epithelial cells derived from organoids obtained from endoscopic biopsies of living human intestine and HIMECs.^99^ The primary cells differentiated into enterocytes, mucus-producing goblet cells and enteroendocrine cells and formed a confluent, polarised cell layer with villi and microvilli structures, exhibiting high similarity in structure and functions to the human duodenum. Additionally, the presence of tight and adherens junctions in both tissue layers was confirmed. Direct comparison of the transcriptomics of the intestinal organoid-derived epithelial chip revealed that it mimics the native duodenum and major functions of the small intestine such as host defence response to infection, digestion, cell proliferation, and response to nutrients better than duodenal organoids. This shows that organoids cultured in static conditions have limitations in recapitulating organ-level functions. Healthy gut organoids or spheroids grown on dynamic microfluidic devices are unfortunately still limited. Regarding disease models the reader is referred to Lim and Park, Ayuso *et al*. and Toley *et al*.^107–109^

### 7.2. Microfluidic liver models

*In vitro* liver models are crucial for the discovery, development and preclinical drug screening.^16^ To develop these models, primary, immortalised and stem cell-derived hepatocytes are used. Due to the difficulty of culturing and loss of function of primary cells, cell lines such as immortalised human hepatocellular carcinoma (HepG2) and human bipotent progenitor (HepaRG) cells are mainly employed.^60^ HepG2 cells exhibit key characteristics of hepatocytes and under certain conditions can form bile canaliculi-like structures between neighbouring cells.^110,111^ As for the HepaRG cell line, cells have the capability to differentiate into hepatocyte-like and biliary cells, show higher CYP450 activity than other hepatocyte cell lines and improved capacity for cholestatic drug screening.^112^ The liver is a highly vascularised organ^113^ and, hence, the development of liver tissue models co-cultured with endothelial cells is essential. HUVECs, immortalised human microvascular endothelial cells (HMEC-1), EA.hy926 cells, LSECs and immortalised bovine aortic endothelial cells (BAECs) are usually used as endothelial cell sources.^30,114–120^ Also, hepatic models often have substitutes for other non-parenchymal cells, such as KCs and stellate cells.^121^ In fact, advanced liver models, like some of those described herein, include immune cells, for instance PBMCs, human histiocytic lymphoma (U937) cells, human monocytic leukaemia (THP-1) cells and human KCs (HK8160 cells).

**Figure 7.**
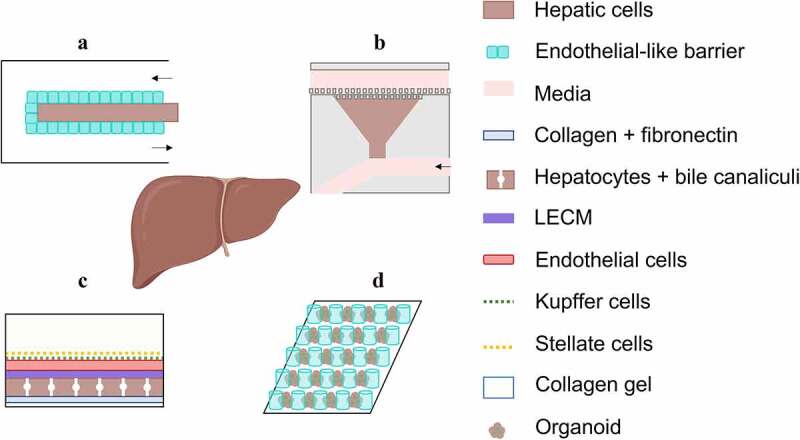
Exemplary schematic illustrations of designs and set-ups for advanced microfluidic liver models. (a) In the model developed by Lee *et al*.^50^ an endothelial-like barrier separates hepatocytes from a flow conducting channel. (b) The triangular design of the model by Shih *et al*.^122^ in combination with multi-row square columns protects the hepatocytes from flow-induced damage and additionally creates a functional acinus model (schematic illustration is based on Shih *et al*.^122^). (c) In the co-culturing set-up of Lee-Montiel *et al*.^117^ which includes hepatocytes with bile ducts separated by a LECM from endothelial, Kupffer and Stellate cells, a perisinusoidal space is mimicked and zone-specific functions are modelled. (d) The design of the flow-through micropillar chip system from Wang *et al*.^123^ allows long-term cultivation and generation of liver organoids from hiPSCs.

The designs of liver microfluidic devices are illustrated in Figure 7anda detailed overview of the *in vitro* models considering the aforementioned cell lines and physiological conditions is given in Table 2. Lee *et al*.^50^ Mazzei *et al*.^148^ Du *et al*.^133^ and Banaeiyan *et al*.^130^ were among the first to design advanced microfluidic liver models. The model developed by Lee *et al*.^50^ comprised a channel seeded with hepatocytes and a flow-conducting channel, which were separated by an endothelial barrier to prevent direct contact of flow with hepatocytes, but still allowed nutrient and oxygen transport by diffusion (Figure 7a).

**Table 2.** Comprehensive overview of the *in vitro* hepatic microfluidic models considering the cultivation method, cell types used, presence of bile ducts and liver zonation, culture period and application. N.D.-Non described.

Scaffold-based *vs*. scaffold-free	Cell types	Mono- *vs*. co-culture	Bile (Yes/N.D.)	Zonation (Yes/N.D.)	Short-term *vs*. long-term	Application	Reference
Device and model development							
Scaffold-based	Primary rat hepatocytes	Mono-culture	N.D.	Yes	Short-term (3 to 4 days)	Zonation-model to study the role of gradients in ischemia-perfusion injury and toxicity.	^124^
Scaffold-based	Primary rat hepatocytes	Mono-culture	Yes	N.D.	Long-term	Cell-cell interactions studies; monitoring hepatocyte functions in a long-term culture.	^125^
Scaffold-based	Primary rat hepatocytes	Mono-culture	N.D.	Yes	Short-term	Drug efficacy and toxicity studies; spatial control of the genetic profile of hepatocytes.	^126^
Scaffold-based	Primary rat hepatocytes	Mono-culture	Yes	N.D.	Short-term (up to 4 days)	*In vitro* model for metabolism and excretion analyses.	^127^
Scaffold-based	Primary rat hepatocytes or cryopreserved primary human hepatocytes	Mono-culture	N.D.	Yes	Short-term (48 h)	*In vitro* model to study liver physiological phenomena and metabolic diseases.	^128^
Scaffold-based	Immortalised human hepatocellular carcinoma (HepG2/C3A) cells or primary hepatocytes	Mono-culture	N.D.	N.D.	Long-term (up to 14 days)	Platform to study drug metabolism by the hepatic and biliary systems.	^129^
Scaffold-based	Human hepatoma derived (HA22T) cells	Mono-culture	N.D.	Yes	Short-term (5 days)	Studies of cell-cell interaction in a liver acinus-like environment.	^122^
Scaffold-based	HepG2 cells or hiPSC-derived hepatocytes	Mono-culture	Yes	N.D.	Long-term (up 21 days)	Drug-induced hepatotoxicity studies.	^130^
Scaffold-based	Cryopreserved human hepatocytes	Mono-culture	N.D.	N.D.	Short-term	Hepatic clearance studies; investigate drug-drug interactions.	^131^
Scaffold-based	HepG2 cells; bovine carotid artery normal endothelial cells (HH cells)	Co-culture	N.D.	Yes	Short-term (5 days)	Drug development; general biological and biochemical studies.	^115^
Scaffold-based	Primary rat hepatocytes; 3T3-J2 fibroblasts	Co-culture	Yes	Yes	Short- to long-term	CYP induction, drug metabolism and drug toxicity studies; investigate the spatial and temporal dynamics of hepatotoxicity.	^132^
Scaffold-based	Primary murine hepatic cells: hepatocytes; LSECs; KCs; HSCs	Co-culture	N.D.	N.D.	Short-term	Investigate cell-cell interactions, cytotoxic metabolism and the inflammatory cascade in a liver sinusoid.	^133^
Scaffold-based	Primary rat hepatocytes; primary or standard endothelial cells	Co-culture	N.D.	N.D.	Long-term (up to 30 days)	Liver toxicology studies.	^114^
Scaffold-based	Dual cell culture model (Primary rat, dog, or human hepatocytes; species-specific rat, dog, or human LSECs); Quadruple cell culture model (aforementioned cell types and species-specific rat, dog, or human KCs and SCs)	Co-culture	Yes	N.D.	Short- to long-term	Species-specific liver-chip for drug toxicity testing and biomarker identification.	^134^
Scaffold-based	HepG2; HUVECs	Co-culture	N.D.	N.D.	Long-term	Drug development and liver physiology studies.	^30^
Scaffold-based	Cryopreserved primary human hepatocytes; EA.hy926 human umbilical vein cell line; U937 cells; LX-2 cells	Co-culture	Yes	N.D.	Long-term (up to 28 days)	Studies of liver physiology, drug safety and drug efficacy under healthy and diseased conditions.	^118^
Scaffold-based	Cryopreserved primary human hepatocytes (Hu8150); cryopreserved HK8160 cells	Co-culture	Yes	N.D.	Long-term	Pharmacokinetics (PK), metabolism, and dose-induced toxicity studies of drugs.	^135^
Scaffold-based	Cryopreserved primary human hepatocytes; human dermal microvascular endothelial cells (HMVEC-D); transformed liver endothelial cells (TMNK-1); HUVECs, EA.hy926 cells; U937 cells; THP-1 cells; LX-2 cells	Co-culture	N.D.	Yes	Long-term (up to 14 days)	Effect evaluation of different concentration of hormones; pilot drug dose studies.	^117^
Scaffold-free	HepG2/C3A cells	Spheroid; mono-culture	Yes	N.D.	Long-term	Predictive drug toxicity assays; modelling of liver diseases.	^136^
Scaffold-free	HepG2 cells	Spheroid; mono-culture	Yes	N.D.	Long-term (up to 14 days)	Physiological and toxicological studies on hepatocytes.	^137^
Scaffold-free	Primary rat hepatocytes; HSCs	Spheroid; co-culture without direct cell–cell contact	No	N.D.	Long-term	Drug screening and toxicity tests; studies of cell-cell interactions.	^17^
Scaffold-free	hiPSCs	Spheroids and Organoids; Mono-culture	Yes	N.D.	Long-term	Platform for engineering stem cell-based organoids with applications in regenerative medicine, disease modelling and drug testing.	^123^
Scaffold-free	Primary human hepatocytes; iHeps; 3T3-J2 fibroblasts	Organoid, co-culture	Yes	N.D.	Long-term (up to 28 days)	Drug screening.	^138^
Scaffold-free	HepaRG cells; HUVECs; PBMCs; LX-2 cells	Organoid; co-culture	Yes	Yes	Short-term (up to 4 days)	Studies on liver physiology, metabolism and underlying molecular processes.	^120^
Drug metabolism studies							
Scaffold-based	Human cryopreserved hepatocytes	Mono-culture	N.D.	N.D.	Short-term	Drug metabolism and clearance studies.	^139^
Scaffold-based	Primary rat hepatocytes	Mono-culture	N.D.	N.D.	Short-term	Predictive studies of metabolic clearance.	^140^
Scaffold-based	Cryopreserved primary human hepatocytes	Mono-culture	N.D.	N.D.	Short-term	Drug screening and toxicity analysis of xenobiotics.	^141^
Scaffold-based	Cryopreserved primary human hepatocytes; EA.hy926 cells; LX-2 cells; U937 cells	Co-culture	Yes	N.D.	Long-term (up to 28 days)	Liver model for drug-related studies and for PK and pharmacodynamic (PD) modelling studies.	^116^
Scaffold-based	Cryopreserved human hepatocytes; non-parenchymal cells	Co-culture	Yes	N.D.	Long-term (up to 10 days)	Drug clearance and metabolism studies.	^142^
Acute and chronic toxicity studies							
Scaffold-based	Primary rat hepatocytes	Mono-culture	N.D.	N.D.	Short-term	Drug toxicity testing.	^143^
Scaffold-based	Freshly isolated human hepatocytes or cryopreserved human hepatocytes or primary rat hepatocytes	Mono-culture	Yes	N.D.	Long-term (up to 14 days)	Acute hepatotoxicity assessment.	^102^
Scaffold-based	HepG2 cells; LX-2 cells; EA.hy926 cells; U937 cells	Co-culture	Yes	N.D.	Short-term	Drug-drug interactions studies and hepatotoxicity testing.	^119^
Scaffold-based	Aggregates of iHeps; HMEC-1 cells; THP-1 cells (differentiated into macrophages)	Co-culture	N.D.	N.D.	Long-term (15 days)	Modelling PK; drug testing.	^144^
Scaffold-based	HepG2-laden collagen and HUVEC-laden collagen	Co-culture	N.D.	N.D.	Long-term (up to 7 days)	Hepatotoxic drug screening.	^145^
Scaffold-free	HepG2/C3A cells	Spheroids; mono-culture	Yes	N.D.	Long-term (30 days)	Drug toxicity assessment.	^146^
Scaffold-free	Primary rat hepatocytes	Spheroids; mono-culture	Yes	Yes	Long-term (up to 2 to 3 weeks)	Acute and chronic drug safety testing.	^147^

Based on these first models, the researchers integrated and studied different parameters (*e.g*., shear stress), cell types and device architectures in order to obtain devices with an environment and functions similar to those of the native liver. For culturing hepatic cells under dynamic conditions, it was found, that *e.g*., vertical flow and the resulting mechanical compaction using a vertical-flow compaction bioreactor array (VCBA) allows to simulate the forces to which hepatocytes are subjected *in vivo*. This improves the functional phenotype of cultured hepatocytes by maintaining tight junctions, forming a polarised cuboidal shape, and a compact morphology even in long-term cultures. Furthermore, higher levels of urea and CYP could be detected and an *in vivo* similar organisation of F-actin was observed in cells (also in monolayer cultures).^125,139,144,149–151^ In addition, some studies have shown the formation of dense tissue structures, including some binucleated hepatocytes, which are structurally and functionally similar to bile ducts.^31,32,102,118,120,123,127,132,135–138,147,152–156^ The replication of bile ducts in *in vitro* liver models is of high importance, since their presence and functionality increases model stability and viability and enables the establishment of long-term models obligatory for chronic toxicity studies.^147,157^ In addition, hepatocyte-specific functions such as albumin and urea synthesis are increased, key genes of drug metabolism of phases I and II are expressed, and induction of enzymes at more physiologically relevant drug concentrations are achieved. In further studies, it was observed, that medium flow increased the expression levels of most of the investigated genes,^150^ led to higher glucose and glutamine consumption, higher ammonia production^151^ and higher CYP activity.^139,144^ However, the prevailing flow conditions require an architectural and geometrical design to prevent unwanted cell death caused by shear stress and to recapitulate metabolic zonation present in the liver tissue. To this end, Shih *et al*. designed a microfluidic device with multi-row square columns and a triangular design of the culture wells to develop a functional and operational acinus model^122^ (see Figure 7b). The innovative architecture not only protected hepatocytes from damage infringed by shear stress during medium perfusion, but also created a semi-circular flow design that imparted a nonlinear gradient to the acinus cell culture area. With higher oxygen and nutrient concentrations in zone 1, but with higher concentrations of soluble factors in zone 3, conditions of the native liver zones could be reproduced in terms of metabolic zonation. However, a higher rate of architecture related cell death in zone 1 could be observed in these types of systems, hence Lee-Montiel *et al*. installed a porcine liver extracellular matrix (LECM) layer into their previously developed human liver acinus micro-physiology system (LAMPS).^117^ The use of materials derived from animals, such as Matrigel®, or hydrogels made from collagen-I have been used as classical representatives of *in vitro* replicated ECM, as they enhance cell adhesion and induce physiological differentiation.^58,59,89–91,97–99^ In addition, non-parenchymal cells, which were separated from hepatocytes by the LECM layer (Figure 7c), were included to mimic the perisinusoidal space (or space of Disse). The improvements in the setup and co-cultivation strategy enabled the establishment of oxygen tension in the acinus across zone 1 and 3 and increased secretion of cytokines and growth factors by non-parenchymal cells. It also allowed modelling of zone-specific functions such as albumin synthesis, nitrogen metabolism, CYP450 activity, and acetaminophen toxicity. Another strategy to dynamically generate metabolic patterns in liver tissue was pursued by McCarty *et al*.^126^ and Kang *et al*.^128^ By actively forcing gradients of various metabolic modulators (*e.g*., hormones), liver tissues from both rats and humans were developed, whose metabolic activities gradually changed throughout the device mimicking gradients of nitrogen, carbohydrate, and xenobiotic metabolism; thus emulating *in vivo* zonation and zonal toxic response.

Although the systems described are well advanced, *in vitro* studies of drug metabolism and extrapolation of metabolic data in drug discovery from *in vitro* systems to human tissues remain challenging and improvements are still needed. In the study of Baudoin *et al*.^140^ primary rat hepatocytes were cultured in a microfluidic device to evaluate hepatic clearance of seven therapeutic molecules. Caffeine, phenacetin, and midazolam showed higher hepatic clearance compared to the *in vitro* data reported in the literature but showed greater similarity to *in vivo* observations. To overcome potential limitations due to the use of animal cell lines and the resulting differences in the species-specific metabolic enzyme CYP450 and its products,^138,158^ such investigations were also conducted with primary human hepatocytes.^116,131,139,141,142^ For this, Prodanov *et al*.^116^ developed an organotypic *in vitro* model to co-culture primary human hepatocytes together with human hepatic stellate (LX-2) cells, KCs (U937 cells) and endothelial cells (EA.hy926 cells) that can replicate the sinusoidal microarchitecture of the liver over a 28-day period. Higher albumin synthesis (synthesis) and urea excretion (detoxification) were observed under fluid flow compared to static cultures, making the model suitable for drug screening studies. Chao *et al*.^131^ also succeeded in long-time culturing of human hepatocytes with high viability, liver cell-specific morphology and cellular metabolic competence under dynamic conditions in HμREL® biochips. Although no decisive influence of the dynamic cultivation strategy on the metabolic activity of hepatocytes could be detected in these studies (*i.e*., similar values for high and medium clearance compounds as under static conditions), a similar experimental setup designed by Prot *et al*.^141^ showed that seven of the ten used metabolites had higher production rates and higher activity of CYP1A2, CYP2C8/9, CYP3A, CYP2D6, and uridine diphosphate glucuronosyltransferase (UGT) compared with conventional culture methods.

Models for testing acute and chronic hepatotoxicity must also be viable and fully functional over an extended period of time, which requires co-cultivation of different cell types.^114,117,118,129,132,134,135,144,145^ HepG2 and HUVECs were cultured under dynamic conditions in the microfluidic device of Mi *et al*.^145^ This allowed the formation of a structurally and functionally biomimetic liver sinusoidal model with an additional channel for the removal of hepatotoxic metabolites. In further studies, the model was extended to include sinusoidal endothelial cells, hepatic stellate cells (HSCs), hepatocytes and macrophages.^118–120^ For example, Deng *et al*.^119^ and Vernetti *et al*.^118^ supplemented their co-cultured liver sinusoidal model with LX-2 cells, endothelial EA.hy926 cells and human monocyte-like U937 cells. The obtained models exhibited physiological distribution of cells and circulation of artificial hepatic blood flow and bile efflux in opposite directions. Thus, active transport of drugs was allowed, synthetic and secretory functions were maintained and enzymatic activities (*e.g*., of CYP450 1A1/2) and drug metabolism sensitivity were preserved up to 28 days. This enabled the investigation of hepatotoxicity and drug interactions of clinically hepatotoxic drugs with high accuracy.^119^ A recent paper by Bircsak *et al*.^144^ reports the development of a microfluidic liver-on-a-chip platform using the OrganoPlate® 2 lane from Mimetas for the co-culture of hepatocyte-like cells differentiated from human induced pluripotent stem cells (iHeps) embedded in collagen (as ECM) with endothelial cells and Kupffer-like immune cells. This physiologically relevant co-culture model showed high cell viability and stable liver function (including albumin and urea production) over 15 days, supported the maturation of iHeps and is, thus, a suitable candidate for accurate and rapid assessment of drug hepatotoxicity.

As an alternative to the scaffold-based cultivation strategies described above, scaffold-free spheroids and organoids can be cultured in individually designed microfluidic devices.^136–138,146,147^ HepG2 hepatocytes embedded in Matrigel® and cultured on an OrganoPlate® device formed spheroids that exhibited bile canaliculi and were viable for 14 days. Albumin and urea production were higher than in static 2D and 3D models, and the spheroids showed high sensitivity to acetaminophen, indicating high potential for predicting drug toxicity.^137^ Yu *et al*. also developed a microfluidic device for culturing rat hepatocyte spheroids to study chronic toxicity in a long-term cell culture under tangential flow.^147^ For this purpose, they designed a perfusion incubator liver chip (PIC) containing a cell culture chamber, a bubble trapping chamber, a heater for temperature control, and a cell culture support. To form spheroids, hepatocytes were enclosed in an ultrathin porous Parylene-C membrane and then transferred to the PIC. This device maintained cell function for two to three weeks, making it possible to repeat administration for more sensitive chronic hepatotoxicity assays.

Further improvement of the functionality of the spheroid-models could be achieved by co-cultivation with, for example, immortalised mouse fibroblasts (3T3-J2 fibroblasts) and HSCs.^17,138^ However, the spherical morphology of multicellular spheroids can limit the supply of nutrients and oxygen, especially to cells in the centre, leading to death or loss of function (*e.g*., dedifferentiation).^159^ To avoid a hypoxic environment and cell death in the spheroids’ core, one strategy is to control the diameter of the spheroid;^160^ nevertheless the oxygen gradient between the core and periphery of the spheroids can be advantageous to mimic the oxygen gradient in the liver lobule. Therefore, a balance between the spheroids diameter and concentration of oxygen must be achieved.^136,146^

Apart from spheroids, human pluripotent stem cells (PSCs) have a strong capacity for self-renewal and self-assembly into organ-like 3D tissues, the so-called organoids. These structures require a suitable microenvironment for their robust formation, which was achieved by Wang *et al*.^123^ (Figure 7d). They were able to culture liver organoids from human induced pluripotent stem cells (hiPSC) in a microfluidic device by adding growth factors. Differentiation took place *in situ*, avoiding the numerous operations required by conventional methods. The average diameter of the organoids increased with differentiation, and they exhibited higher viability under dynamic than under static conditions. These liver organoids showed cellular heterogeneity that included hepatocytes and ductal structures. In addition, dose- and time-dependent acetaminophen toxicity was detected in the 3D liver organoid model, indicating that this system can provide a potential platform for preliminary drug testing.

## 8. Microfluidic *in vitro* models of the gut-liver interaction

**Figure 8.**
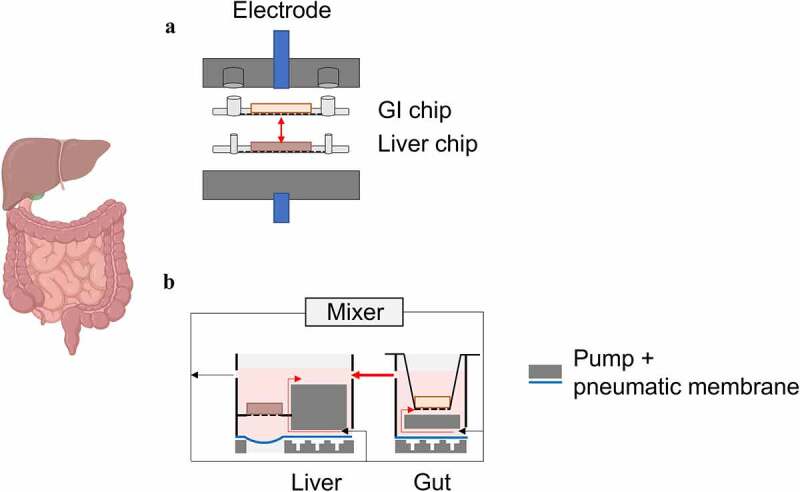
Schematic illustrations of multi-organ chip approaches. (a) In the gut-liver-on-a-chip system developed by Esch *et al*.^161^ human primary liver cells and Caco-2 cells are cultured on the porous membranes of the single chips. Once maturation is observed, the chips are connected to set up the multi-organ model with integrated TEER electrodes; (as published by Esch *et al*.^161^). (b) In the automated multi-organ design of Tsamandouras *et al*.^166^ the gut and liver compartments are connected in series via peristaltic pumps; by installing a mixing chamber, the flow of the culture medium can be portioned to mimic physiological conditions.

Given that after oral administration the intestine and liver are closely linked and serve important functions, there has been increasing interest in the design of multi-organ *in vitro* systems, such as multi-organ-on-chip.^161–163^ These devices incorporate two or more tissue compartments, which are connected through microfluidic tubes into a single device. The integration of several tissues into a single platform can be divided into static, semi-static and flexible approaches. The static method accommodates organs/tissues into chambers connected by a single, unidirectional fluid flow. The different types of cells are seeded on the chambers and supplied with a universal culture medium.^164,165^ Due to the unchangeable configuration of the chip, the interorgan connection occurs in a predefined disposition, where only upstream organs interact with the downstream ones. In the event of organ/tissue failure, it becomes impossible to alter the failed tissue compartment and thus the chip becomes unusable. Semi-static approaches generally embody cell culture inserts with permeable membranes interconnected by microfluidic channels and may additionally incorporate typical microfluidic components such as pumps and sensors.^164,165^ The tissue in each chamber can be pre-cultured according to their needs prior to incorporation, and thereafter circulation of culture medium through the compartments occurs to mimic systemic blood circulation. Flexible systems are designed so that the order, number and type of tissues can be freely established. Briefly, several individual single-organ chips are interconnected by microfluidic tubing forming multiple, modular configurations. Each of these individual organ-on-chips can be cultured separately with the organ-specific culture medium until it reaches the required level of maturation or differentiation. Once the conditions for the integration of the various organs are met, the compartments are interconnected, and the organs cultured collectively.^164,165^ To mimic the intestine-liver interaction, a gut-liver-on-a-chip with integrated transepithelial electrical resistance (TEER) electrodes based on the flexible approach was developed by Esch *et al*., which combines primary cells of human origin to construct the liver tissue, and Caco-2 cells for the gut^161^ (Figure 8a). Each tissue was cultured onto a single-organ chip until maturation was observed and subsequently gut and liver chips were combined to set up the multi-organ-on-chip. The entire platform included a top layer containing the fluidic channel for the intestinal tissue, a porous membrane with Caco-2 cells, a layer for the fluidic channel of the liver tissue, a 3D scaffold for hepatocytes and non-parenchymal cells and finally a base at the bottom. Both single-organ chips were designed to incorporate a fluidic circuit. The microfluidic tube of the intestinal chip perfused the apical side of the intestinal epithelium while that of the liver chip perfused the basolateral side of the intestinal epithelium and the liver chamber. Thereby, the latter channel resembles the systemic circulation of the body and the culture medium supplied to the apical chamber resembles the fluids present in the human intestinal lumen. Rocket platforms in combination with a customised valve mechanism enabled a pumpless system with physiologically relevant and unidirectional flow so that shear-sensitive tissues could be included. It was shown that the adoption of unidirectional flow in the gut-liver system resulted in a stable barrier integrity of the intestinal epithelium during the 14 days of co-culture with the liver, which was not the case with bidirectional flow. The liver tissue maintained high levels of metabolic activity and reacted to toxic substances because of CYP enzyme activity. Despite all the advantageous features of this system such as maintaining cell co-culture up to 14 days, being modular, influence of unidirectional flow, and the embedded TEER electrodes, the multi-organ lacks optical accessibility and an automated and continuous culture medium flow. Conversely, Tsamandouras *et al*. have developed an automated gut-liver-organ-on-chip that uses peristaltic pumps to circulate fluid through the tissue chambers^166^ (Figure 8b). This platform includes five different chambers, *i.e*., the first for integrating a cell culture insert with a permeable membrane in which intestinal tissue is cultured, the second for a 3D-perfused liver, the third and fourth for two other organs of interest, and the fifth for a mixing chamber reproducing the systemic circulation compartment. Intestinal tissue was composed of human intestinal epithelial (Caco2-BBe) cells, HT29-MTX cells, and primary monocytic dendritic cells. Liver tissue comprises a 3D co-culture of human primary cryopreserved hepatocytes and KCs. Prior to their transfer to the multi-organ-on-chip, the cells were pre-cultured for a defined time. To mimic *in vivo* organ physiology and flow partitioning as closely as possible, the intestine and liver compartments were connected in series, and the output of the mixing chamber was partitioned such that 75% of the culture medium flowed into the intestine and the remaining 25% into the liver. Since the universal culture medium in the gut-liver circuit flows through the basolateral side of the gut insert and *via* the liver and mixing chamber, and the medium on the apical side of the gut tissue is supplied by a different culture medium, it was possible to mimic oral and intravenous administration. The former is achieved by drug application into the apical part of the intestinal tissue insert and the latter by addition of a drug directly into the mixing chamber. The results showed potential for studying bioavailability, drug-drug interactions, effects of repeated drug exposure and metabolite accumulation and investigation of low clearance compounds. However, the type of 2D culture and the cell variability used to mimic the intestinal tissue did not allow to recreate the metabolic function of the intestine *in vivo*. Thus, the proposed multi-organ-on-chip had the tendency to under-predict the hepatic clearance. Cell heterogeneity and 3D cell culture appear to improve the prediction of pharmacokinetic parameters and drug toxicity on gut-liver-organ-on-chip. Recently, several drugs such as acetaminophen and the prodrug mycophenolate mofetil were tested with similar models based on Caco-2, HT29 cells and primary hepatocytes, respectively.^167,168^ Through the comprehensive evaluation of the mechanistic model, such as identification ability of the structural model, the parameters and a global sensitivity analysis, a robust experimental design was established, and an estimation of the *in vitro* pharmacokinetics (PK) parameters was achieved. These studies suggest that with the combination of device architecture and different cultivation methods (*e.g*., 3D cell culture, cell heterogeneity and fluid flow), possible synergistic results can be obtained.^161,169,170^ However, an important aspect to be considered in the development of multi-organ-on-chip especially for PK prediction is the scaling of the different organs towards each other. A study with the aim to produce a human gut-liver chip to investigate the first-pass metabolism of various drugs revealed discrepancies between the PK parameters (peak time, peak concentration, half-life time) obtained and the known PK parameters from humans. These discrepancies decreased when the gut absorptive area and liver cell volume were changed, which improved the PK profile in the chip.^171^

## 9. Challenges and future perspectives

After reviewing the literature, it becomes clear that cells cultured in micro-sized chambers under microfluidic conditions, experience physiological conditions close to those occurring on native tissues. Indeed, studies showed that fluid shear stress enhanced differentiation and mucus formation of intestinal epithelial cells and improved the phenotype and CYP activity of hepatic cells.^94,99,139,144,172^ To accomplish this, the flow rate applied to each system needs to be optimised in order to obtain a shear rate value that effectively stimulates the cells. These factors and their adoption strongly depend on the cell source. Regarding the intestine, in most cases, Caco-2 cells are used and combined with HT29-MTX cells. Although Caco-2 cells are known to recapitulate several physiological and pathophysiological functions of the human intestine, they are immortalised cells originally isolated from a human tumour. As an alternative, human intestinal tissue explants have been explored, but they are difficult to culture. To mimic the ECM, Matrigel®, a matrix extracted from the Engelbreth-Holm-Swarm mouse sarcoma is mainly used. However, the applicability of Matrigel® is limited due to its complex, poorly defined and variable composition. Variations in mechanical and biochemical properties within batches of Matrigel® can lead to lack of reproducibility. In addition, Matrigel® cannot be manipulated physically or biochemically, making it difficult to fine-tune the matrix to promote the desired cell behaviour and achieve specific biological outcomes. Accordingly, alternative xenogen-free and chemically defined alternatives should be considered.^173^ Another important aspect in the intestine that should be taken into account is the microbiota. The human intestine hosts a myriad of distinct microbes that contribute to metabolism and digestion and influence the endocrine system.^174,175^ Indeed, the heterogeneity and complexity of the gut microbiota can affect, either directly or indirectly, drug absorption, distribution and metabolism.^176,177^ Thus, the implementation of the most relevant intestinal microorganisms and/or metabolites are of great relevance and potentially can lead to breakthrough advances in this area.

Concerning the development of hepatic tissue there are also discrepancies in the cell sources used. Primary hepatocytes, which are preferentially used, are difficult to isolate and often incompatible, but express intrinsic features of the liver.^178^ In comparison, hepatocytes derived from stem cells produce albumin and urea but are expensive and require specific induction factors. Moreover, liver models incorporating biliary ducts and capable of simulating the *in vivo* cell-cell and cell-matrix interactions are still lacking. This could recently be circumvented and improved through the use of bioprinting.^179,180^ Bioprinting is a fairly new technology that allows 3D printing of living cells, layer by layer. At present, this process is still being researched and is expected to be revolutionary in the future. The use of this technology for the manufacture of gastrointestinal tissue models^181–183^ allows the geometric properties of the tissue construct to be controlled through precise patterning and positioning of multiple cell types, obtaining heterocellular microenvironments similar to native tissues, while maintaining a low risk of cross-contamination.^146,184^ Only a few studies were found for the liver.^185^ Chang *et al*.^186^ bioprinted hepatocytes embedded in alginate into a microfluidic device, obtaining an *in vivo*-like 3D structure with improved differentiated function and a higher amount of synthesised urea compared to 2D hepatocyte monolayer culture. By bioprinting liver spheroids in a microfluidic device, the bioactivity of the hepatocytes could be kept constant for 30 days and a hepatotoxicity response similar to that of *in vivo* and *in vitro* models could be recorded.^146^ Massa *et al*. created a vascularised 3D liver tissue model by bioprinting HepG2/C3A cells encapsulated in a gelatin methacryloyl (GelMA) hydrogel.^187^ To create the central vessel for mimicking vascularised tissue, a sacrificial agarose fibre was printed. After the removal of this fibre a hollow microchannel was formed (diameter of 100–1000 µm) and endothelial cells were seeded, creating a monolayer of endothelial cells that allows the exchange of media, nutrients, oxygen, and even drugs with the tissue, thus making it possible to test physiological drug diffusion and toxicity. Pharmacological damage to the endothelial barrier, and hence the assessment of the suitability of the system for testing hepatotoxicity, was evaluated by perfusing the system with acetaminophen. The results showed that the metabolic activity of endothelial cells decreased, proving that the model can recapitulate the hepatotoxicity of the drug. Moreover, the presence of an endothelial monolayer increased the metabolic activity of HepG2/C3A cells. A more advanced and complex system was developed by Lee *et al*., in which a biliary channel was introduced in order to mimic bile canaliculi, which represent important structures for drug metabolism *in vivo*.^180^ They 3D-printed a microfluidic device with dual flow to supply nutrients to the cells present in the vascular channel (top channel) and to remove bile acid and waste products from the bile channel (bottom channel). The two channels were divided by a printed porous membrane, on which HepaRG cells, encapsulated in a liver decellularised extracellular matrix (dECM) bioink, were printed. Then, to fully simulate a liver sinusoid, HUVECs embedded in gelatine were printed on the HepaRG layer. The results showed that the cells were correctly positioned, the HepaRGs differentiated into hepatocyte- and biliary like cells, and the HUVECs exhibited adherens junctions. In addition, the dual flow channel induced higher expression of cytokeratin 19 (CK19), multidrug resistance-associated protein 2 (MRP2), organic anion transporting polypeptide 1B3 (OATP1B3), albumin, alpha-fetoprotein (AFP) marker, and the mature liver-specific gene transthyretin (TTR). All these observations may be attributed to the efficient formation of a biliary system and thus to the removal of waste products and bile acid, as well as higher hepatocyte functionality. Subsequently, the established model was successfully used to sensitively assess *in vitro* toxicity of acetaminophen, hence demonstrating potential to screen potential drug candidates. Despite the progress towards bioprinting technology in combination with microfluidic devices, the materials in which cells are embedded still lack cell guidance properties, physical and biochemical stimuli capable of inducing changes in the cellular phenotype after bioprinting and experience difficulties in sterilisation.^188^ Therefore, new materials are required and essential to achieve a bioink capable of supporting cell growth and with mechanical and physical properties equivalent to those of native tissues. Additionally, the bioprinter’s resolution must be improved to facilitate the printing of complex single cell structures such as capillary networks and a balance between fabrication time and mechanical strength should be established. The latter is due to the fact that fabrication time affects the mechanical strength of the printed structure, such that, lower fabrication time enhances the mechanical strength due to earlier crosslink. On the other hand, it increases the shear stress between the nozzle and the bioink, damaging the cells. Hence, it would be advantageous to use four dimensional (4D) printing, a method in which the materials reshape in a pre-programed and active manner over time, through an external stimulus.^189^ Again, it is noteworthy that the advances in bioprinting do not fulfil all the requirements to reproduce a realistic environment for cells given that maintaining appropriate conditions for long-term tissue culture is also needed. Moreover, the majority of the microdevices are fabricated with PDMS, which is flexible, gas-permeable and crystal clear, enabling microscopic imaging. However, it may absorb small drug molecules, possibly yielding misleading results.^190^ Some studies manufactured devices with materials that show minimal absorption of hydrophobic compounds to ensure that the drug loss is negligible, and the exposure can be controlled;^135,166,191^ nonetheless, the number of studies using these materials is still scarce and needs further research. In addition, it is critical to integrate or improve existing physical, chemical, and biological sensors in cell culture devices to monitor external stimuli in a non-invasive manner in real time, in order to achieve adequate quality control of the conditions in the culture chambers.^192,193^ Minimising experiments performed on a trial and error basis is important to counteract significant waste of consumables and meet Goal 12 of the 2030 Agenda for Sustainable Development.^194^ For instance, to fight the waste in flow rate optimisation experiments, researchers can perform computational fluid dynamics (CFD) simulations to determine the shear stress that the cells are subjected at a given flow rate. This will allow them to perform fewer laboratory experiments, saving time and resources.

Another important challenge is the long development time of microfluidic devices, which includes the phases of design, prototyping in the laboratory and validation before potential mass production is possible.^195^ Thereby, the first two phases define the feasibility and suitability of the device, which need to consider a variety of complex factors such as material selection, prototyping process, as well as the physiologically relevant design concept to ensure the process scalability, biocompatibility and manufacturing precision. The error rate in the third development phase, i.e., the validation phase, can be rather high due to the required integration of multiple materials and different microfabrication processes. In particular, the validation of systems without standard protocols poses a challenge in terms of quality control and process optimisation.^196^ Apart from the device development, the accessibility and the device handling represent another challenge requiring specific expertise and training. This includes, for example, the integration of the relevant hardware (*e.g*., pumps) into commercial microfluidic devices by the end user. With regard to cell culture-related handling, the poorly controllable mode of cell seeding is noteworthy. In conventional cell culture formats (*i.e*., commercial well plates and cell culture inserts), controllable and reproducible cell seeding can be performed directly onto the membrane. By contrast, cell seeding under fluid flow hampers the control of homogeneous attachment of cells to the designated sites in the microfluidic device. This affects the required cell number, the cell density that can be achieved, and consequently the time to obtain an intact barrier.^197^ Given these hindrances, end-users might prefer to continue with their conventional practices and instruments.^198^

Another limitation of the current platforms is that cell samples are collected by dismantling the chips which can damage the sample and contaminate the environment, hampering the experimental procedures. In addition, microfluidic devices are often not compatible with standard experimental protocols.^199^ For instance, traditional assays such as flow cytometric analysis and gel electrophoresis-based techniques cannot yet be readily integrated into microfluidic devices. It is necessary to improve or even modify protocols to provide a complete biological characterisation.^200^ Accordingly, standardisation in terms of features, materials, production processes and biological test methods still needs to be addressed.^201^ In order to overcome the accessibility issue, the future microfluidic devices will require automated manufacturing and handling combined with a user-friendly interface.^202,203^

Reconfigurable microfluidic devices have emerged as potential solutions to overcome the aforementioned problems. In brief, this concept involves the assembly and integration of individually designed and fabricated modular components into a single microfluidic device according to the end-user’s requirements.^204^ It can combine the advantages of traditional static devices with those of microfluidic platforms, *i.e*., giving the user the ability to directly seed the cells on the membrane and perform standard assays, while applying shear stress to cells. This can be achieved by assembling, disassembling and reconfiguring the basic modules into the microfluidic device, which results in switching between static and dynamic conditions.^197^ A major advantage of such a modular design is that users can test components separately before assembly. Furthermore, faulty components can be replaced quickly without considerable cost and long development times, as is the case with monolithic devices.^204^ Despite these advantages, fluid leakage can occur in the fluidic interconnections of individual modules, which poses a design challenge that must be overcome.

With technological advances, new opportunities arise for the most diverse areas. An example of this are the *in silico* methods such as machine learning which portray an interesting asset to predict drug toxicity and adverse effects after compound exposure by combining this method with transcriptomic, genomic or proteomic data.^205^ The utilisation of accurate mathematical models allied to *in vitro* models constitute a helpful method to reduce animal testing as well as to decreasing the costs in the preclinical test phase. Some studies already demonstrated progresses in developing *in silico* models with this goal in mind, however, they still face some limitations such as poor data quality, insufficient accuracy and lack of information about the relationship between substructures and chemical toxicity, which is helpful to optimise the drug compounds.^205–210^ Technological and scientific breakthroughs are also expected to pave the way for developments in personalised organ-on-chips. This field propose the production of systems that test the patient’s individual response to drugs *in vitro*, which will result in ethical and reliable data concerning drug efficacy, potency, and tissue toxicity as was well as reducing predictability differences between *in vitro, in vivo* and clinical trials. In other words, these devices will have added value for personalised treatment and prevention strategies, probably contributing to the evolution of medicine.^211^

## 10. Conclusion

Numerous attempts are being made to improve *in vitro* gut, liver, and gut-liver microfluidic models by considering physiological factors such as fluid flow, shear stress, separation of the vascular and parenchymal compartments, significant mechanical influences, and tissue-tissue interfaces at the organ level. These devices facilitate drug toxicity screening and allow conclusions to be drawn about the PK of orally administered drugs. However, the complete structure, functional mechanisms, and specific physiological environmental conditions such as gastric and intestinal fluids, enzymes, complex microbiota and the respective metabolites are still lacking. Furthermore, experimental results obtained using these microfluidic devices are often not reproducible, in part owing to the lack of available quality control and performance criteria.^212–214^ In order to keep pace with advances in medicine, to evaluate rapidly and efficiently new drug candidates and test drug delivery systems, these models need to be further adapted and a number of limitations to be addressed. Overall, there is still a long way to go to overcome these challenges and to develop a human gut-liver model or a complete body-on-chip that can accurately predict the body’s response to a drug and its toxicity.
